# Spatial distribution and structural discrimination of quorum sensing metabolites in *Pseudomonas aeruginosa* by MALDI-MSI

**DOI:** 10.1039/d6an00245e

**Published:** 2026-06-30

**Authors:** C. Kuik, S. W. G. van Hoogstraten, L. Mayrginther, J. J. C. Arts, M. Honing, B. Cillero-Pastor

**Affiliations:** a Maastricht MultiModal Molecular Imaging Institute (M4i), Maastricht University Maastricht The Netherlands b.cilleropastor@maastrichtuniversity.nl; b Department of Orthopaedic Surgery, Laboratory for Experimental Orthopaedics, CAPHRI, Maastricht University Medical Centre Maastricht The Netherlands; c Department of Biomedical Engineering, Orthopaedic Biomechanics, Eindhoven University of Technology Eindhoven The Netherlands; d Department of Pathology, GROW – School for Oncology & Reproduction, Maastricht University Medical Centre+ Maastricht The Netherlands; e Department of Cell Biology-Inspired Tissue Engineering, MERLN Institute for Technology-Inspired Regenerative Medicine, Maastricht University Maastricht The Netherlands

## Abstract

Bacterial biofilms are complex bacterial communities embedded within an extracellular polymeric substance (EPS) that protects the cells and increases survival. Once established on an orthopaedic implant, a biofilm can cause difficult-to-treat and severe infections. *Pseudomonas aeruginosa* is of particular concern due to its strong biofilm-forming capability. Within biofilm communities, quorum sensing (QS) networks regulate biofilm formation, virulence, and intercellular communication. This is achieved through the production of signalling molecules, QS metabolites. In *P. aeruginosa*, quinolones such as the *Pseudomonas* quinolone signal (PQS) and its precursor 2-heptyl-4-quinolone (HHQ) are among the key regulators. However, although these signalling molecules regulate essential functions such as biofilm development, virulence regulation, and population-level communication, our understanding of their spatial distribution within *P. aeruginosa* biofilms is still limited. QS metabolites, also referred to as autoinducers, help in the regulation of biofilm formation and dispersion. These small molecules, including quinolones, often have an isomeric counter partner which is not involved as autoinducer. Matrix-assisted laser desorption/ionization mass spectrometry imaging (MALDI-MSI) offers a label-free approach for the spatial detection of these metabolites directly within biofilms. To differentiate isomeric quinolones, such as PQS and 2-heptyl-4-hydroxyquinoline-*N*-oxide (HQNO), tandem MS (MS/MS) and trapped ion mobility spectrometry (TIMS) coupled to time-of-flight (TOF) mass spectrometry are employed in this study, with the addition of salt, sodium chloride, to enhance sodium adduct formation. This integrated approach provides spatially resolved insights into QS metabolite organisation, demonstrating an improved strategy for distinguishing isomeric species within bacterial biofilms and advancing our understanding of bacterial communication and potential anti-biofilm targets.

## Abstract 2

Spatial mapping of Pseudomonas aeruginosa quorum-sensing metabolites using MALDI-TOF and TIMS separation enables differentiation of quinolones in biofilms.

## Graphical abstract

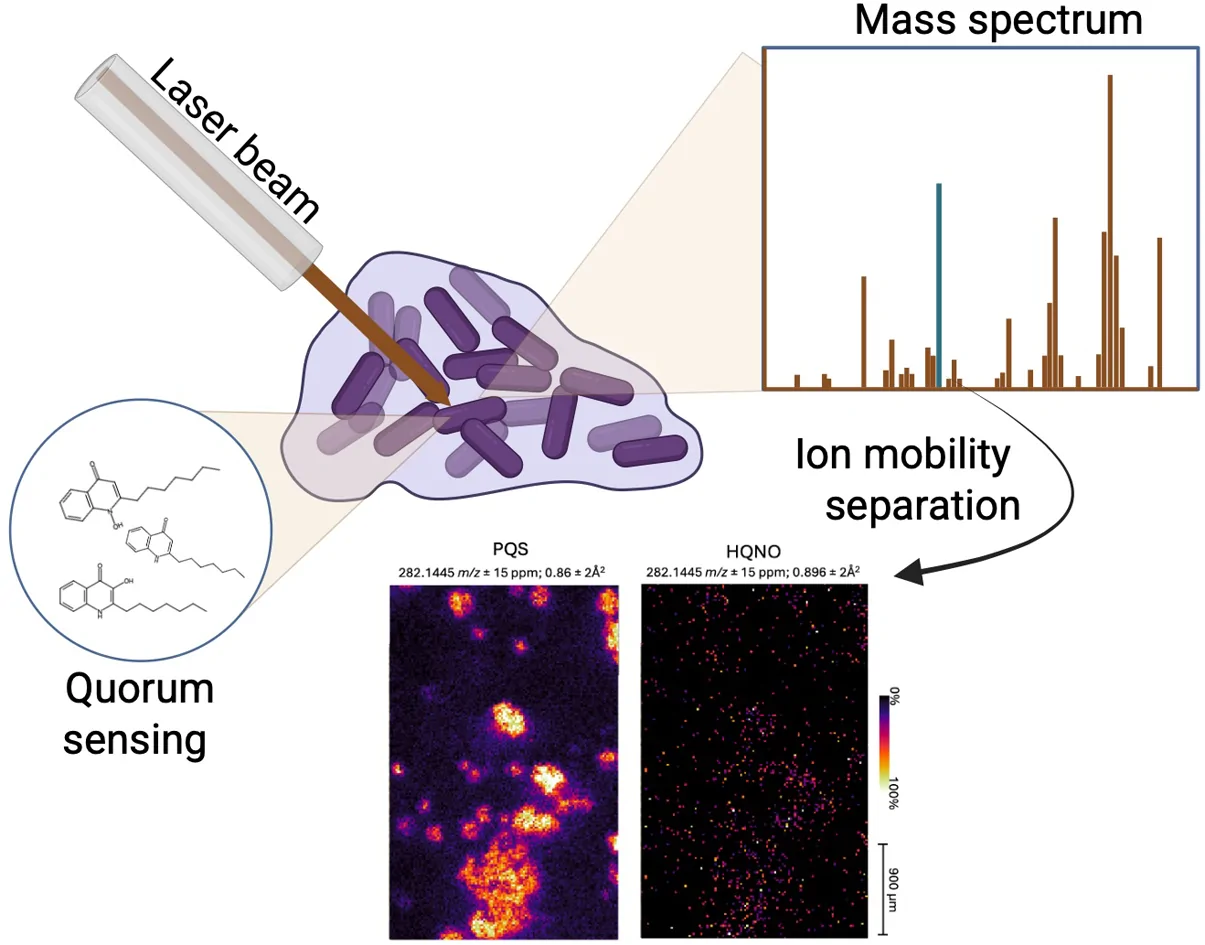

## Introduction

Biofilms are a bacteria stage in which cells are embedded in an extracellular matrix when they colonise biotic or abiotic surfaces. The matrix is composed of water and an EPS, mainly consisting of extracellular DNA, polysaccharides and various proteins.^1,2^ When a biofilm is established on a medical implant, such as a joint replacement prosthesis, it can lead to severe infections. *Pseudomonas aeruginosa* is of particular concern due to its strong ability to form biofilms on implant surfaces and their high antibiotic resistance.^3^

Metabolites secreted by the biofilm are part of an intra- and inter-bacterial communication system known as QS.^4^ It allows bacteria to adapt from the lifestyle of an individual cell to that of a community. In addition, it is essential in biofilm formation, dispersion and metabolic exchange between bacteria, regulating cell density and biofilm growth.^4^ This process is mediated by the sensing and production of QS metabolites excreted by the bacteria, reflecting the cell population density in an EPS.^5^ Autoinduction is achieved when the accumulation of the QS molecules reaches a threshold concentration, generating a positive feedback loop.

In *P. aeruginosa*, QS is a well-structured communication system consisting of four different QS pathways: the Las, Rhl, PQS, and integrated QS systems.^4,5^ The Las system utilises the molecule 3-oxo-S12-homoserine lactone (HSL) as an autoinducer (AI), which binds to the LasR receptor.^6^ This system has multiple functions, including stimulating the production of siderophores, molecules responsible for maintaining the structural integrity of the biofilm. LasR stimulates the formation of Rhl, PQS and IQS. The LasR system is a crucial component in the formation of virulence factors, such as rhamnolipids. Rhamnolipids are formed when host defence cells are detected and can lyse macrophages and neutrophils. The PQS system is specific to *Pseudomonas* bacteria and regulates over 90 genes in *P. aeruginosa*, including regulating the virulence factor pyocyanin. The leading AI is PQS, and its precursor is 2-heptyl-4-quinolone (HHQ).

Regulation of QS can offer novel antibiofilm strategies.^1^ However, there is limited detailed information about the complex QS systems and metabolite regulation within biofilms. A better understanding of the local QS presence can lead to the development of new strategies to prevent the formation, maturation and dispersal of biofilms.^3^ Furthermore, *P. aeruginosa* produces more than 50 other quinolones functioning as secondary metabolites.^5^ Besides acting as AIs, they also play essential roles as antibiotics against competing species and as virulence factors regulating host responses. In *P. aeruginosa*, the quinolones can be classified into three groups: the PQS, AQ, and 2-alkyl-4(1*H*)-quinolone *N*-oxides (HQNOs). All these classes are produced as mixtures with saturated and unsaturated C_5_–C_11_ alkyl chains. Detailed information about the *P. aeruginosa* QS system and metabolites is limited, and new QS molecules are still being discovered.^5^ Therefore, more in-depth insight into bacterial communication in biofilms is needed, as it can lead to the development of new anti-biofilm strategies.

Previously, MALDI-MSI has been used to detect QS metabolites and other quinolones in bacterial biofilms.^7–12^ MSI is a label-free imaging technique that allows for the direct visualisation of molecular species, including metabolites, on various surfaces. MALDI is the most widely used MSI ionization technique, in which a laser scans over the sample, impacting the surface to enable absorption and ionisation of the molecules disposed in a specific matrix on the surface.

MALDI-MSI demonstrated its usefulness in detecting QS metabolites and quinolones in various bacterial biofilms, as well as in the interactions of multiple bacterial species.^7–10^ Using this method, QS molecules were localised, identified and tracked over time, gaining knowledge about the molecular distribution of the molecules and their development during biofilm formation. Pitchapa *et al.* detected the distribution of *N*-acyl-homoserine lactone-related metabolites and quinolones over time in *P. putida* biofilms.^7^ Here, a difference in formation between quinolones and *N*-acyl-homoserine lactone-related metabolites was observed, showing a spread of quinolones in mature biofilms and localised *N*-acyl-homoserine lactone-related metabolites in early-stage biofilms.

Previously lactone-related metabolites were spatially resolved to study biofilm formation. However, sample preparation of bacterial biofilms for MALDI-MSI remains challenging. Agar-based methods typically require the agar-grown biofilm to be transferred onto a glass slide suitable for MSI analysis. This process can compromise the structural integrity of the biofilm. This limitation can be addressed by cultivating the biofilm directly on appropriate glass slides, which preserves the spatial structure and more accurately reflects an infection situation.

Additionally, biofilms formed by *P. aeruginosa* encompass multiple isomeric quinolone metabolites. For instance, the key QS metabolite PQS shares the same molecular sum formula as 2-heptyl-4-hydroxyquinoline-*N*-oxide (HQNO), a secondary metabolite and virulence factor. These isomeric quinolones cannot be distinguished by MS1 alone.

To overcome this, two strategies were investigated. First, MS/MS was used to study the fragmentation pattern of the isomers in detail, aiming to distinguish between the PQS and HQNO based on their fragmentation. Secondly, TIMS coupled to a TOF mass spectrometer system was employed, allowing the separation of ions not only based on *m*/*z*, but also by their gas-phase mobility. Previously, the use of salt adduct ions has been proved to enhance the separation of structural and stereoisomers.^13^ This salt adduct separation can occur since the adduct formation can stabilize specific gas-phase conformers of the isomeric molecules, causing subtle differences in their structural conformation and configuration, next to the collision cross-section (CCS), resulting in improved ion mobility separation.

In this study, we demonstrate the spatially resolved detection of QS metabolites in *P. aeruginosa* using MSI with TIMS-TOF, while preserving the native spatial distribution of the biofilm by growing it directly on compatible ITO slides.

## Materials and methods

### Materials

ITO slides were purchased from Delta Technologies (Loveland, CO, USA). 2,5-Dihydroxybenzoic acid (DHB) was purchased from Sigma-Aldrich (St Louis, MI, USA). Methanol (MeOH), ethanol (EtOH), and chloroform (CHCl_3_) were purchased from Biosolve (Valkenswaard, NB, The Netherlands). Purified HHQ, PQS, C4-HSL and oxo-C12-HSL standards were purchased from Merck (Darmstadt, Hesse, Germany); HQNO was purchased from TargetMOL (Boston, MA, USA).

### Biofilm cultivation

ATCC 27853 was inoculated in 5 ml of Luria–Bertani broth and shaken at 37 °C for 16–24 hours; the stock was diluted to 10^7^ CFU ml^−1^ in Luria–Bertani broth. 2 ml of the diluted stock and 1 ml of Luria–Bertani broth were added to sterile ITO slides inside a well plate; the well plates were transferred to a humidified box. The biofilms were left to grow for 17 days at 37 °C; the medium was refreshed every 7 days. When a slide reached its designated age, it was removed and washed twice with 2 ml of Milli-Q water. The bottoms of the slides were washed with 70% ethanol. The biofilms were grown in triplicate, each consisting of a 14-day-old biofilm. The slides were desiccated and transferred to a freezer at −80 °C for at least 24 hours before analysis.

The biofilm formation was verified using scanning electron microscopy (SEM) using a JEOL SEM (JSM-IT200).

### Standard preparation

Standards were prepared for HHQ, PQS, HQNO and oxo-C12-HSL. The metabolite standards were diluted in 7 : 3 MeOH : H_2_O to final concentrations of 0.1–1000 µM. The matrix was prepared as 20 mg ml^−1^ DHB, 3 : 7 MeOH : H_2_O. The matrix solution was sonicated for 10 minutes and filtered through a 0.45 µm filter (VWR International, PA, USA). Both the standard solution and the MALDI matrix were mixed in a 1 : 1 ratio and 1 µl was spotted onto an MTP ground steel target plate (Bruker Daltonics, Bremen, BRE, Germany).

### MALDI-MSI

To dehydrate the biofilms before MALDI analysis, the slides were desiccated for 1 hour before applying the matrix. The DHB matrix solution (12 mg ml^−1^ DHB in 1 : 2 MeOH : CHCl_3_) was coated onto the slides using an HTX M3+ sprayer (HTX Technologies, Chapel Hill, NC, USA). The following parameters were used for matrix application: the temperature was set to 50 °C, the flow rate was set to 120 µl min^−1^, the velocity was set to 1200 mm min^−1^, the spacing was set to 3 mm, the N2 pressure was set to 10 psi, the number of passes was set to 15, and spraying was performed using a C–C pattern. Optical images of the DHB-coated slides were taken using a Reflecta MF5000 (Reflecta, Eutingen im Gäu, BW, Germany).

MALDI-TOF analysis was performed using a TimsTOF fleX MALDI-2 instrument (Bruker Daltonics, Bremen, BRE, Germany) equipped with two Nd/YAG lasers at wavelengths of 355 and 266 nm for MALDI and MALDI-2, respectively. The MSI experiments were performed in positive ion mode, with the mass range set between 100 and 1000 *m*/*z* and a lateral resolution of 30 µm^2^. Calibration was performed using red phosphorus with a standard deviation of no more than 5 ppm error. Regions of interest were created, which were kept the same between all measured samples. The MALDI laser was set to 1 burst of 150 shots at 10 000 Hz. The laser power was optimized prior to acquisition and kept constant during MSI measurements, by not exceeding an intensity of 1 × 10^4^. Variations between samples reflect adjustments to compensate for differences in biofilm height (focus) to maintain a consistent ion signal intensity within the detector's dynamic range. The TOF reflector was operated with a plate offset of 30 V, and the Deflect 1 Delta was set to 70 V. The funnel 1 RF was kept at 250 Vpp. The ionization energy was set to 5 eV and a collision RF of 1200 Vpp was applied.

The raw datasets were imported into SCiLS Lab (Bruker Daltonics, Bremen, Germany) and normalised by TIC. Peak picking was performed using mMass with an *s*/*n* tolerance of 3, an intensity threshold of 0.1, and a peak height of 50%. The resulting peak-picked data were then imported into SCiLS Lab for unsupervised segmentation analysis. Segmentation was performed using the bisecting *k*-means clustering algorithm with a correlation distance metric, grouping pixels based on spectral similarity to identify regions with distinct molecular signatures within the biofilm samples.

### TIMS analysis

For the TIMS metabolite separation of standards and TIMS-TOF imaging, the 1/*k*_0_ start was set to 0.50 V s cm^−2^ and the 1/*k*_0_ end was set to 1.2 V s cm^−2^. A ramp time of 150 ms and a 100% duty cycle were employed. The MALDI laser was set to 150 shots at 1000 Hz per pixel.

TIMS calibration was performed using nitrogen as the drift gas, and the CCS values were derived from the measured 1/*k*_0_ values using the Agilent ESI-L Low Concentration Tuning Mix according to the manufacturer's instructions.

The use of NaCl to improve ion mobility-based separation was evaluated for both the metabolite standards and biofilm samples. For the metabolite standards, 1 mg ml^−1^ NaCl was added directly to the DHB MALDI matrix solution. For MSI analysis of the biofilm, NaCl was applied by spraying the sample with five layers of a 1 mg mL^−1^ NaCl solution prepared in 70 : 30 (v/v) acetonitrile/water. Each layer was manually applied using a thin layer chromatography (TLC) sprayer, with a one-minute drying interval between applications. A total amount of 0.14 mg of NaCl was sprayed onto the slide.

### Tandem mass spectrometry

MS/MS spectra were acquired using collision-induced dissociation (CID) fragmentation. The fragmentation was performed using nitrogen as a collision gas with the mass tolerance set to ±1. The charge applied included 15, 20, 25, 30, 35 and 40 eV, to obtain the desired fragmentation patterns.

## Results and discussion

### Detection of *Pseudomonas aeruginosa* metabolites

Since the ITO slide surface may influence bacterial behavior and does not fully replicate *in vivo* conditions, the biofilm formation on the ITO slides was assessed and confirmed using SEM (Fig. S1). This approach provides a practical compromise to enable MALDI-MSI analysis while preserving the biofilm structure. The observed morphology was consistent with typical *P. aeruginosa* biofilm formation Before MALDI-MSI analysis, the detection of five metabolite standards, including the QS metabolites HHQ, PQS, C4-HSL, oxo-C12-HSL and HQNO as isomeric counter partners of HHQ, was evaluated by regular MALDI. These included the QS quinolone metabolites HHQ and PSQ, along with two *N*-acyl-homoserine lactone-related metabolites. The secondary metabolite HQNO was included due to its structural similarity to PQS. Fig. S2 shows the MS1 spectra of the metabolites and Table 1 provides an overview of the selected metabolites and their sum formulae, masses, and chemical structures.

**Table 1:** Overview of selected metabolites for MALDI standards analysis. Information regarding the selected metabolites is listed, including their sum formulae, mass in g mol^−1^, and chemical structures

	Molecule	Molecular class	Sum formula	Mass (g mol^−1^)	[M + H]^+^/[M + Na]^+^	Chemical structure
HHQ	2-Heptyl-4-quinolone	AQ	C_16_H_21_NO	243.1623	244.1701/266.1521	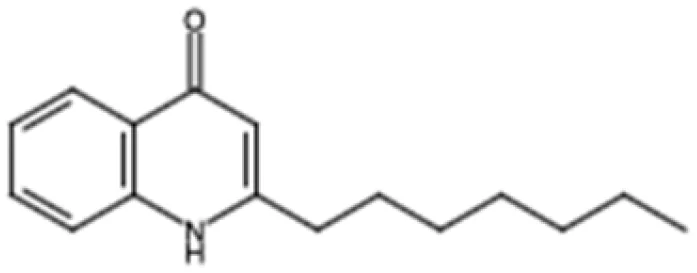
PQS	2-Heptyl-3-hydroxy-4-quinolone	PQS	C_16_H_21_NO_2_	259.1572	260.1651/282.1570	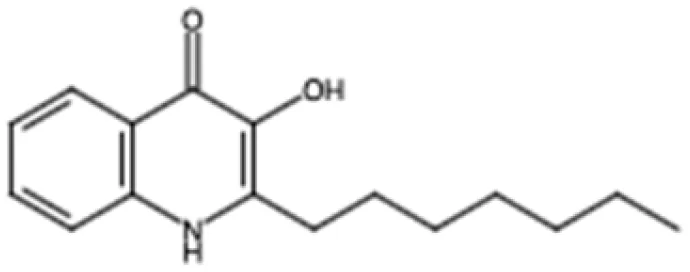
HQNO	2-Heptyl-4-hydroxyquinoline-*N*-oxide	AQNO	C_16_H_21_NO_2_	259.1572	260.1651/282.1570	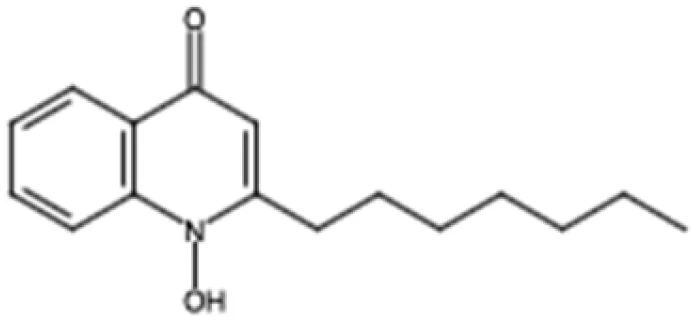
Oxo-C12-HSL	*N*-3-Oxo-dodecanoyl-l-homoserine lactone	HSL	C_16_H_27_NO_4_	297.1940	297.2019/320.1838	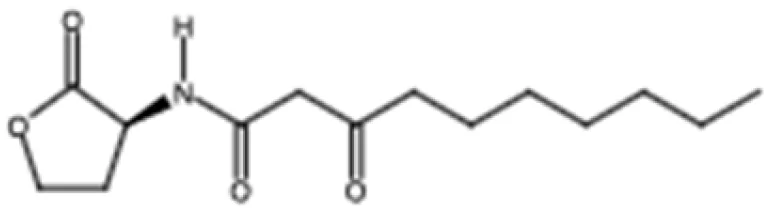
C4-HSL	*N*-Butyrylhomoserine lactone	HSL	C_8_H_13_NO_3_	171.0896	172.0974/194.0933	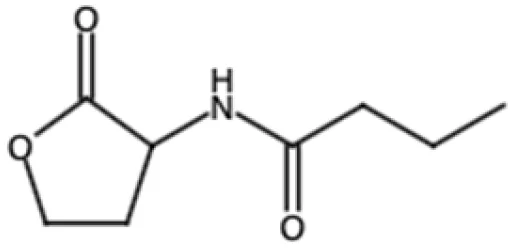

All compounds were detected except for *N*-acyl-homoserine lactone-related metabolite C4-HSL, not detected at any of the concentrations. This low-molecular-weight molecule (171.19 Da) lacks prone-to-ionise functional groups, such as basic amines or acidic carboxyls, which typically facilitate protonation or deprotonation during MALDI ionisation. The lactone ring is neutral and will not easily ionise under MALDI conditions. Additionally, the nitrogen in the amide group is conjugated, making it less basic and therefore less likely to protonate efficiently.^14^

An effective strategy for improving the ionisation and detection of the C4-HSL metabolite is through chemical derivatisation. This approach involves modifying molecules by introducing a readily ionisable functional group, thereby enhancing the ionisation efficiency by MALDI. An example of this was demonstrated by Kim *et al.*, where a lactone-related metabolite was derivatised with Girard's reagent T, significantly improving the sensitivity by up to 60 000 times, as indicated by the lower limit of detection with MALDI.^15^

### Investigation of tandem mass spectrometry fragmentation of quorum sensing molecules

For the structural identification of metabolites, and more specifically for isomeric molecules, various MS/MS and ion mobility spectrometry (IMS) methodologies can be used. In more detail, the fragmentation pathways of isomeric structures are highly related to the molecular conformation, which can also be derived from the so-called CCS or the arrival time distribution (ATD).

**Fig. 1:**
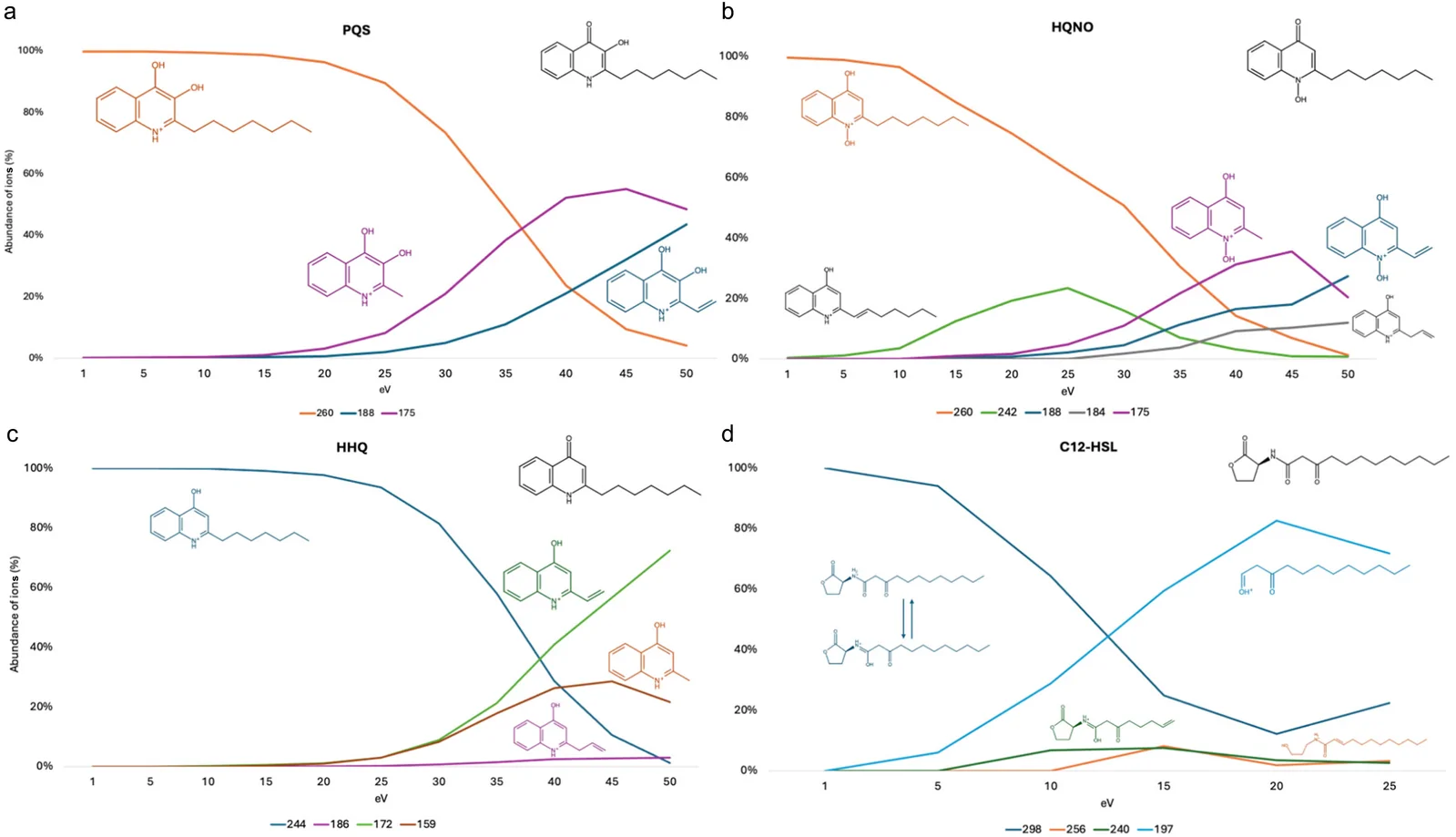
Fragments of (A) PQS, (B) HQNO, (C) HHQ and (D) C12-HSL, as functions of CID energy of QS metabolites, including their suggested fragments.

In a first step, the MS/MS spectra of the protonated ions of PQS, HQNO, HHQ, and C12-HSL were acquired at collision energies of 15, 20, 25, 30, 35, and 40 eV. This experiment was performed to investigate if the fragmentation pattern of the molecules allows both isomers to be distinguished. The resulting fragment intensities, plotted as a function of collision energy, are shown in Fig. 1.

**Fig. 2:**
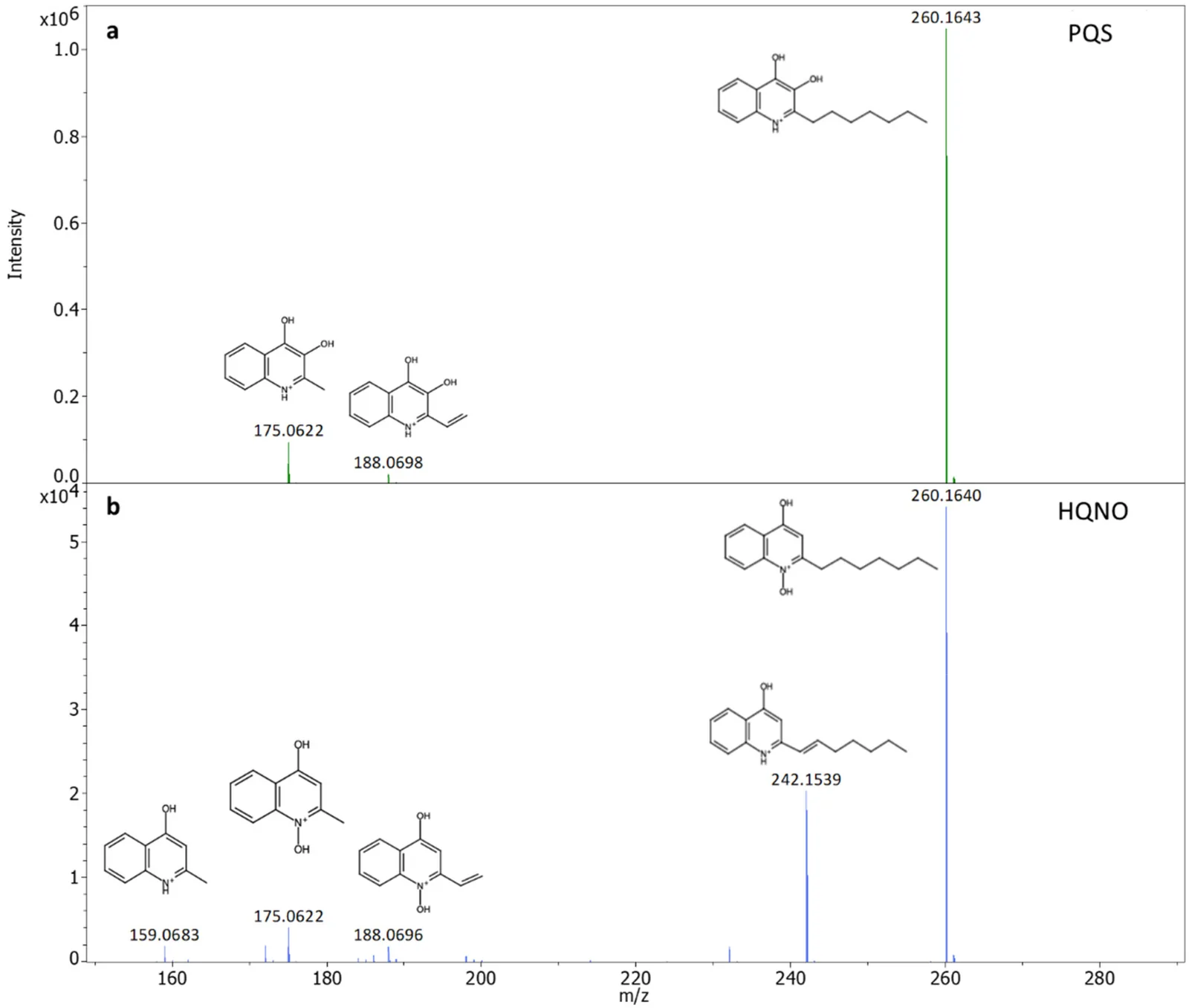
MS/MS spectra of (a) PQS and (b) HQNO, including fragment annotation.

The fragmentation patterns of PQS and HQNO at 40 eV (shown in Fig. 2) reveal that the MS/MS spectra of the [M + H]^+^ of the isomeric ions differ significantly. HQNO predominantly underwent water loss from the protonated molecule, whereas PQS displayed no water loss. Both molecules produced two major product types, including the formation of cations by neutral losses of the alkyl chain and radical cation fragments,^16^ resulting in the predicted radical cation fragment of *m*/*z* 175 and a cation fragment of *m*/*z* 188. However, the relative intensities of these fragments varied considerably. For PQS, the illustrative fragment ions at *m*/*z* 188 and *m*/*z* 175 appear as low as at 15 eV, while for HQNO they become prominent at approximately 20 eV. This is despite the HQNO fragments already being detected at 10 eV, significantly lower than the 20 eV and even 30 eV needed for PQS and HHQ.

Furthermore, HQNO appears to exhibit two distinct fragmentation pathways: one arising from neutral losses of the alkyl side chain and the formation of radical cation fragments from the protonated molecule, and the other originating from the parent ion undergoing water loss. In contrast, PQS and HHQ display only the fragmentation pattern arising from the neutral losses. Differences in the fragmentation pathways and energy thresholds facilitate the distinction of even the isomeric QS metabolites, enabling selective quantification through specific MRM transitions.

### Separation of PQS and HQNO using ion mobility separation

**Fig. 3:**
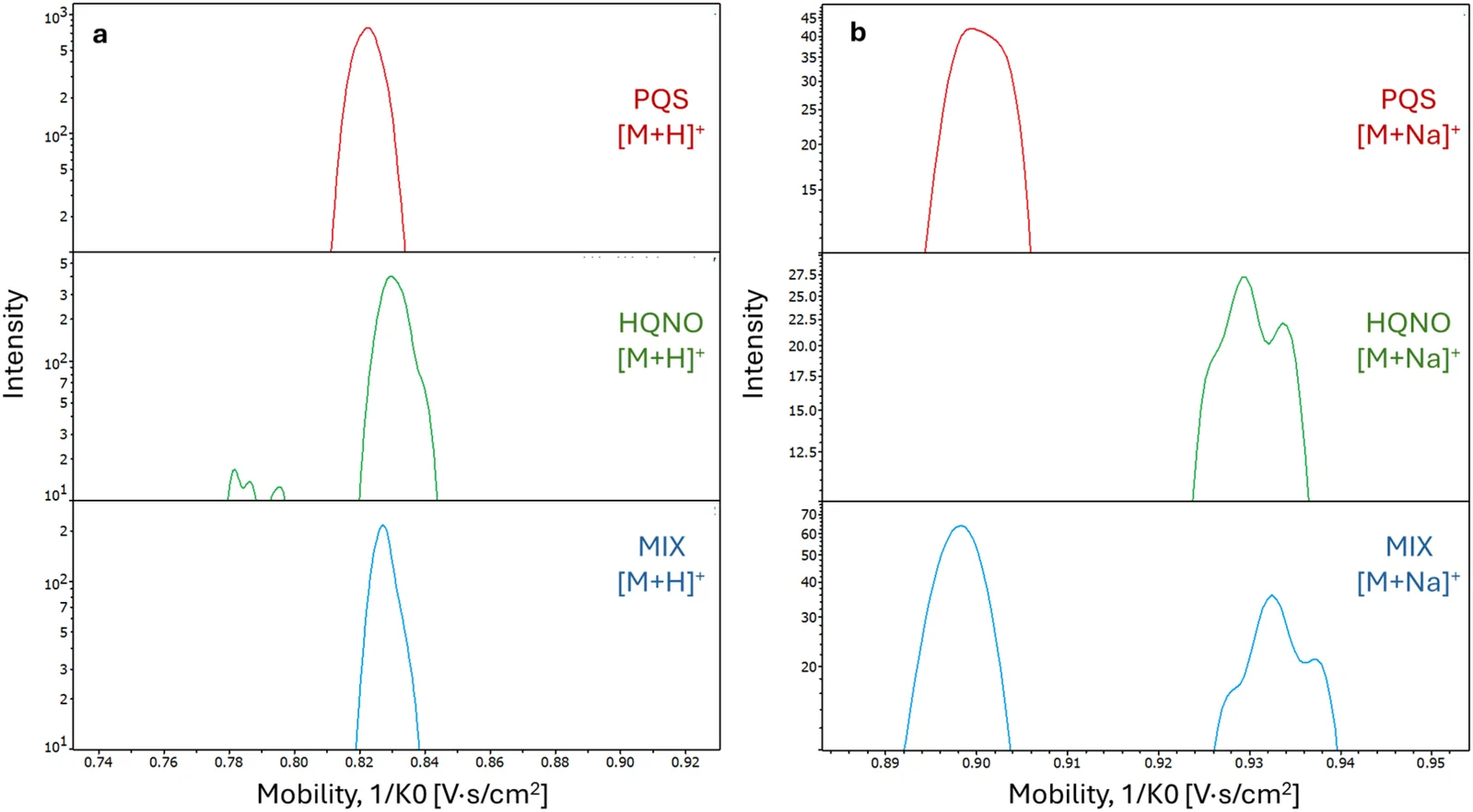
Separation of PQS, HQNO, and a mixture of both using IMS. (a) Mobiligrams of PSQ, HQNO and a mixture when analysing the [M + H]^+^ adduct. (b) Separation of PQS and HQNO when separating the sodiated form.

To further investigate the separation of the isomeric QS metabolite, the IMS separation of PQS and HQNO was investigated by varying the ramp time, ion mobility range, and accumulation time. The detection of the protonated species [M + H]^+^ using TIMS-TOF, shown in Fig. 3a, did not result in successful separation of the two isomers. However, upon addition of NaCl, the sodium-adducted ions ([M + Na]^+^) compounds could be effectively separated. A clear mobility shift in between the two quinolones was observed and is shown in Fig. 3b, showing the separation of PQS and HQNO alone and in a mixture.

Improved separation upon sodium adduction arises from stabilization of different gas-phase conformers of the isomeric molecules, leading to measurable differences in their CCS values and, consequently, in their ion mobilities. Several metal cations, including Na^+^, Li^+^, Ag^+^, and Cs^+^, have previously been employed to enhance IMS separation of isomeric compounds.^13,17^

Previous studies have demonstrated that metal adducts bind at different functional sites on isomeric analytes, inducing distinct gas-phase conformations and enabling their separation by IMS.^13,17^ In the case of PQS and HQNO, the sodium ion most likely coordinates to the oxygen atom *via* ion–dipole interactions.^17^ Because the position of the oxygen functionality is the primary structural difference between the two isomers, sodium coordination forces each molecule into a distinct, separable orientation.

The longer drift time observed for the sodium-adducted HQNO ion is consistent with a more elongated gas-phase structure when sodium is bound to the oxygen atom.^18^ Furthermore, peak broadening of PQS was observed for the sodium-adducted ions, along with additional separation of HQNO (Fig. 3b). This behavior may result from multiple possible sodium coordination sites in HQNO due to the presence of two oxygen atoms, leading to the formation of multiple gas-phase conformers and, consequently, multiple mobility peaks.^17,18^ Such effects provide additional insight into the complexation and binding mechanisms of these molecules.

Recently, the distribution of isomeric quinolones in *P. aeruginosa* was studied by MALDI using fragment ions formed by MS/MS of the molecule of interest. Using this method, the authors showed a difference in the distribution of targeted PQS and HQNO metabolites.^19^ Combining the MS/MS approach with TIMS separation for salt adducts can give comprehensive insights into the chemical composition and, ultimately, distribution. TIMS analysis can be used to separate isomers of a metabolite simultaneously during a TIMS experiment, and MS/MS can be employed to identify molecules of interest.

### Molecular imaging of quorum sensing metabolites in *P. aeruginosa* biofilms

Next, the spatial distribution of the quorum-sensing metabolites was investigated using MALDI-MSI, including the distribution of the isomeric HHQ and HQNO. The *P. aeruginosa* biofilms were grown directly on an ITO glass slide to minimise sample handling and therefore disruption of the sample's structure.

**Fig. 4:**
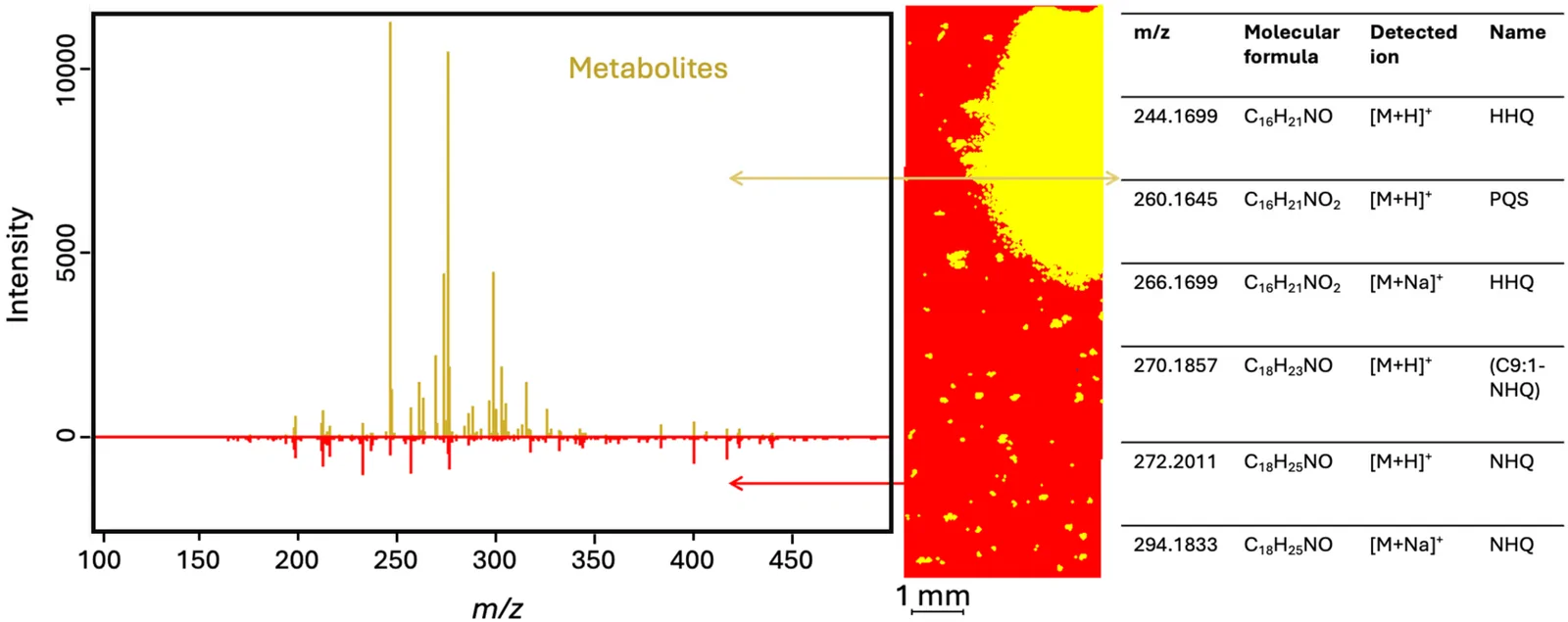
Visualisation of segmentation analysis of a 14-day-old biofilm. The segmentation reveals two distinct regions, corresponding to the metabolites highlighted in yellow. The identification of the QS metabolites is confirmed by MS/MS.

When investigating the distribution of the molecules within the bacterial biofilm, two distinct areas can be localised. Segmentation analysis of the full biofilm sample dataset was used to inspect the molecular signature of regions, revealing areas with high metabolite abundance. The segmentation data are shown in Fig. 4, which includes the mass spectra of segmented regions and a list of the six most abundant ions in the metabolite region. The MS/MS spectra used for the identification, including the predicted fragmentation, of these molecules are shown in Fig. S3.

**Fig. 5:**
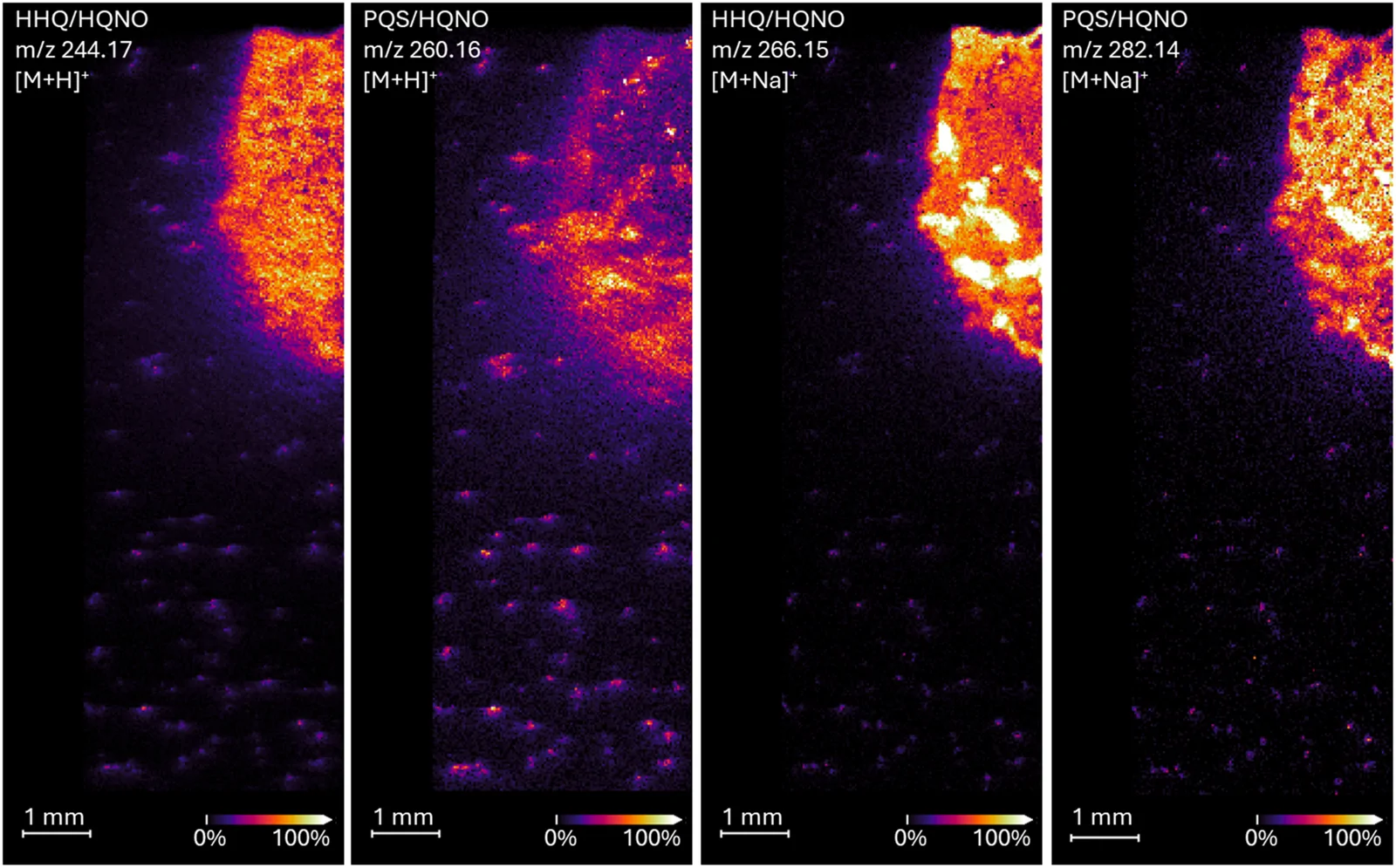
Distribution of QS metabolites in the *P. aeruginosa* biofilm by MALDI-MSI. The distribution of four ions is visualized corresponding to HHQ, HQNO, and PQS.

The spatial distribution of QS metabolites HHQ and PQS in a 14-day-old biofilm is shown in Fig. 5. The optical image of this sample is provided in Fig. S4. Distinct differences in ion distribution are observed in the ion images. The image at *m*/*z* 244.17 is correlated with either the QS metabolite precursor HHQ or the secondary metabolite HQNO, which can undergo loss of a hydroxy group, resulting in the same molecular mass as HHQ. In contrast, *m*/*z* 260.16 displays a notably different spatial distribution, suggesting the presence of protonated PQS or HQNO.

Previous studies, focusing on the detection of QS metabolites in *P. aeruginosa*, primarily focused on improving the sensitivity and coverage of molecular detection, rather than on the separation of isomeric compounds.^7,9,10^ However, numerous structurally related and isomeric metabolites naturally occur within *P. aeruginosa* and can exhibit markedly different, and sometimes opposing, biological functions within the biofilm.^5^ To address this analytical and biological complexity, MALDI-IMS-MSI combined with salt addition was employed in this study to enable the separation and spatial mapping of isomeric QS compounds directly within the biofilm.

### Imaging of PQS and HQNO by MSI and IMS using salt adducts

**Fig. 6:**
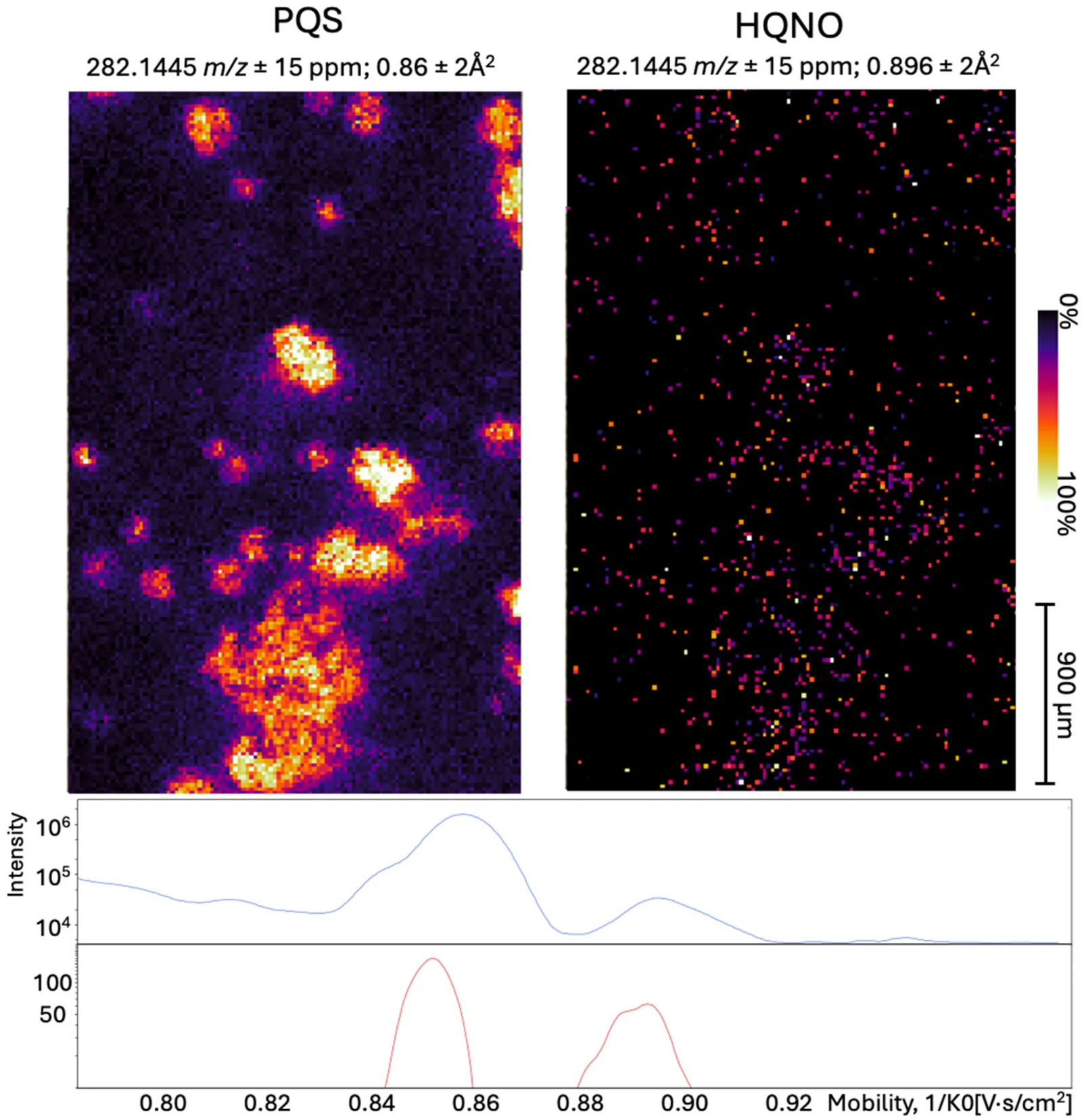
Distribution of HQNO and PQS in the *P. aeruginosa* biofilm analysed by MALDI-IMS-MSI using salt adducts. The lower panel shows 2 mobiligrams: the blue one corresponds to the imaging data and the bottom red mobiligram corresponds to the standard analysis of PQS (left) and HQNO (right).

To differentiate PQS and HQNO in biofilm imaging, MALDI-MSI was performed using IMS separation. Consistent with previous findings, the addition of salt enabled the separation of these isomers. Therefore, the biofilm sample was sprayed with a salt solution prior to MALDI-IMS-MSI analysis, allowing the spatial analysis of the isomers within the *P. aeruginosa* biofilm, shown in Fig. 6. The 1/*k*_0_ values observed in the imaging mode corresponded directly to the values obtained from separate analysis of PQS and HQNO. The dominant signal originated from the molecule with a 1/*k*_0_ value of 0.86, corresponding to PQS. These results confirm that the detected ion from the ion image with an *m*/*z* of 282.14 mainly originates from the QS metabolite PQS and not HQNO. A slight difference in 1/*k*_0_ values and peak shapes compared to standards is visible. This likely arises from the nature of imaging data, where signals are accumulated across many pixels, resulting in a broader ion population. In addition, low-level biofilm molecules or matrix-related signals may contribute to these minor variations.

This spatial distribution is consistent with the work of Shen *et al.*, who performed 3D mapping of QS molecules in 24 h old *P. aeruginosa* biofilms and demonstrated that HQNO was primarily localised in the inner layers of the biofilm, whereas PQS was enriched in the outer layer.^19^ Furthermore, Baig *et al.* used MS/MS fragmentation with SIMS to track the distribution of QS metabolites in 7-day-old *P. aeruginosa* biofilms, revealing a higher intensity of HQNO fragments.^20^ As the current MALDI-IMS-MSI measurements mainly probe the biofilm surface, it is therefore highly plausible that mainly the outer biofilm layer was sampled, explaining the strong dominance of the PQS signal in the present study.

The ability to directly discriminate PQS and HQNO *in situ* highlights the analytical strength of combining sodium-adduct formation with TIMS-resolved MALDI-MSI. This approach overcomes a major analytical challenge in quinolone imaging to distinguish isomeric QS metabolites and provides a platform for investigating spatial QS signaling within complex biofilm architectures. Such spatially resolved information is essential for a deeper understanding of how QS signaling is organized within biofilms and may ultimately contribute to the identification of spatially targeted anti-biofilm intervention strategies. Nevertheless, variations in biofilm thickness and sample flatness can influence laser focus and local ionization efficiency during MSI acquisition. Although TIC normalization was applied to reduce signal variability across the dataset,^21^ residual intensity differences related to biofilm heterogeneity may remain, and the use of isotopically labelled quorum sensing metabolites as internal standards could further improve correction for local ionization effects in future studies.^22^

Next to quinolones, other *P. aeruginosa* metabolites, including pyocyanin ([M + H]^+^) and rhamnolipids (Rha-C10–C10 and Rha-C10–C12, primarily observed as [M + Na]^+^), were detected, supporting the biological relevance of MSI in biofilm spatial molecular analysis (Fig. S5).

## Conclusion

In this study, the distribution of QS metabolites in *P. aeruginosa* biofilms was investigated using MALDI-MSI. Separation of the isomeric metabolites PQS and HQNO was achieved by incorporating IMS with the addition of salt, which enabled separation of their sodium adducts. Subsequent MALDI-IMS-MSI analysis provided spatial localization, revealing that PQS was the predominant isomer detected within the biofilm. These findings demonstrate the utility of MALDI-IMS-MSI for resolving structurally similar QS metabolites and highlight PQS as a major contributor at the biofilm surface.

## Author contributions

CK and SvH designed and performed the experiments, analyzed the data and wrote the manuscript. JJCA, MH and BCP designed the experiments, supervised the writing and approved the latest version.

## Conflicts of interest

The authors declare no competing interests.

## Supplementary Material

AN-151-D6AN00245E-s001

## Data Availability

The data supporting this article have been included as part of the supplementary information (SI) and in Metaspace (https://metaspace2020.org/project/kuik-2026). Supplementary information is available. See DOI: https://doi.org/10.1039/d6an00245e.
