# Supplementary material for: Spatial distribution and structural discrimination of quorum sensing metabolites in Pseudomonas aeruginosa by MALDI-MSI
Source: Analyst. 2026 Jun 30. Online ahead of print. doi: 10.1039/d6an00245e (PMC13403072; doi:10.1039/d6an00245e)
Supplement: AN-OLF-D6AN00245E-s001 [file AN-OLF-D6AN00245E-s001.pdf]

This journal is © The Royal Society of Chemistry 2026

Supplementary information

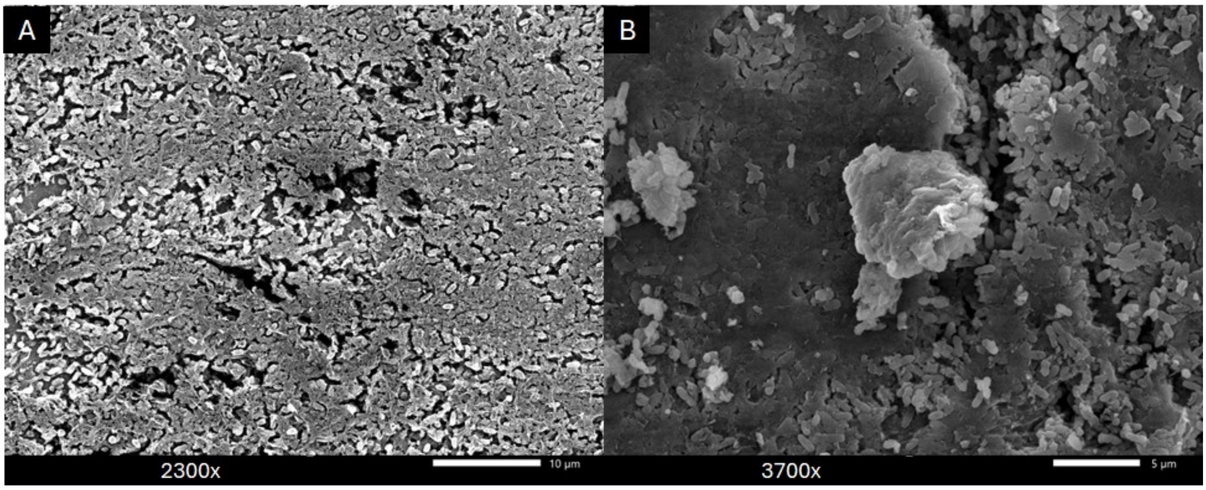

Figure S1. SEM imaging of *P. aeruginosa* showing a 2300x zoom (A) and a 3700x zoom (B).

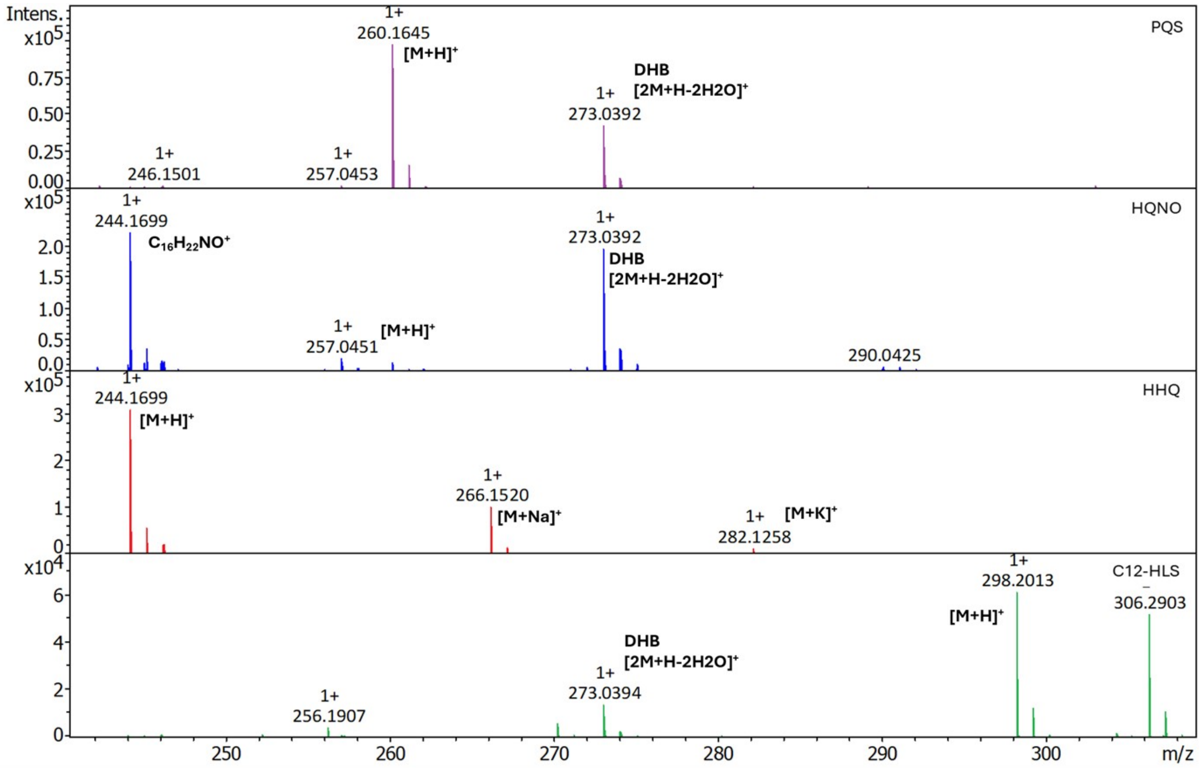

Figure S2. MS1 spectra of QS metabolite standards.

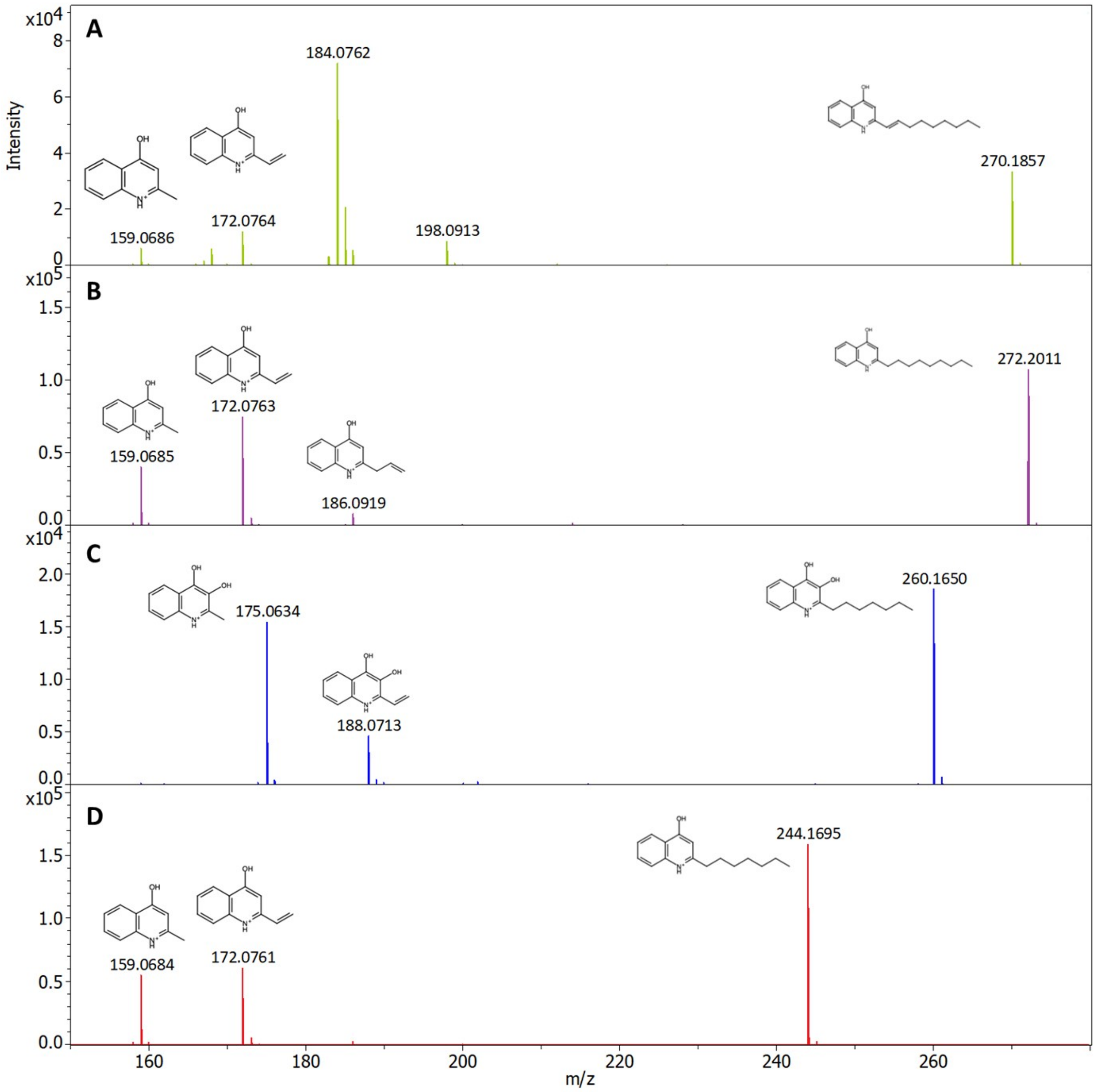

Figure S3. MSMS spectra of A) 270.1857, B) 272.2011, C) 260.1650, and D) 244.1695 on *P. aeruginosa* biofilm.

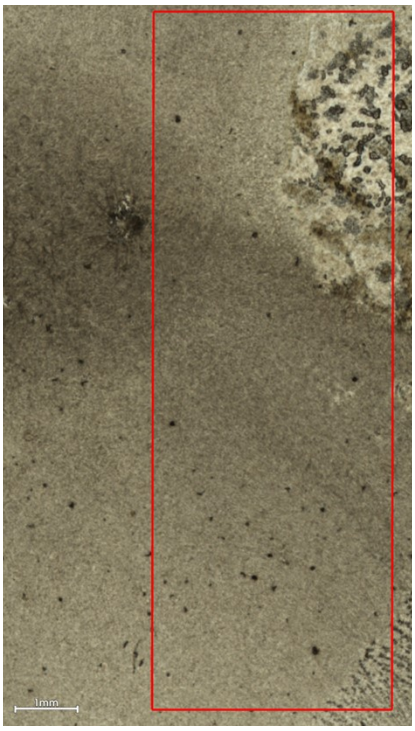

Figure S4. Optical image of 14-day old *P. aeruginosa* biofilm.

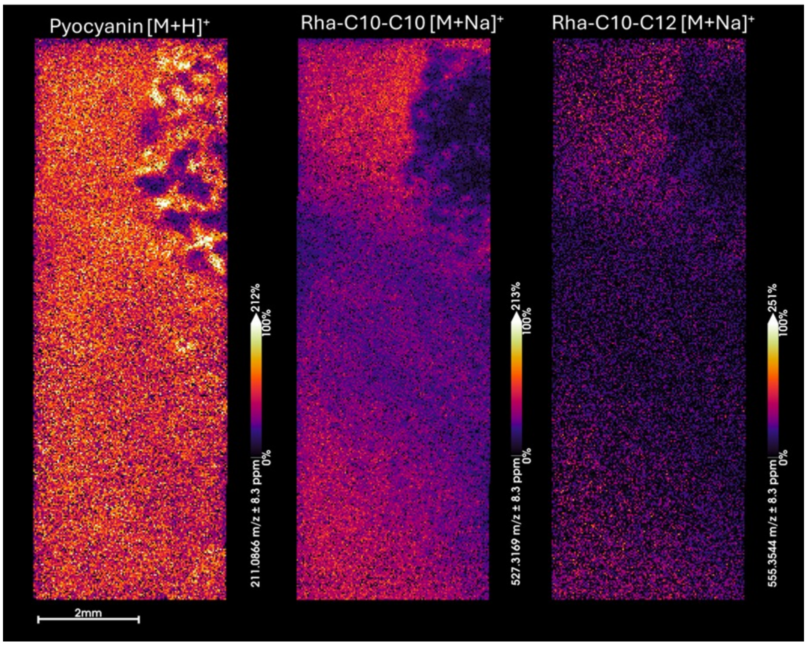

Figure S5. Distribution of pyocyanin and two rhamnolipids in *P. aeruginosa* biofilm analysed by MALDI-IMS-MSI using salt adducts.
